# SARS-CoV-2 Spike Protein Downregulates Cell Surface α7nAChR through a Helical Motif in the Spike Neck

**DOI:** 10.1021/acschemneuro.2c00610

**Published:** 2023-02-06

**Authors:** Tommy S. Tillman, Qiang Chen, Vasyl Bondarenko, Jonathan A. Coleman, Yan Xu, Pei Tang

**Affiliations:** † Department of Anesthesiology and Perioperative Medicine, University of Pittsburgh, Pittsburgh, Pennsylvania 15260, United States; ‡ Department of Structural Biology, University of Pittsburgh, Pittsburgh, Pennsylvania 15260, United States; § Department of Pharmacology and Chemical Biology, University of Pittsburgh, Pittsburgh, Pennsylvania 15260, United States; ∥ Department of Physics and Astronomy, University of Pittsburgh, Pittsburgh, Pennsylvania 15260, United States; ⊥ Department of Computational and Systems Biology, University of Pittsburgh, Pittsburgh, Pennsylvania 15260, United States

**Keywords:** α7 nicotinic acetylcholine receptor, α7nAChR, SARS-CoV-2, spike protein, mRNA vaccines, long COVID

## Abstract

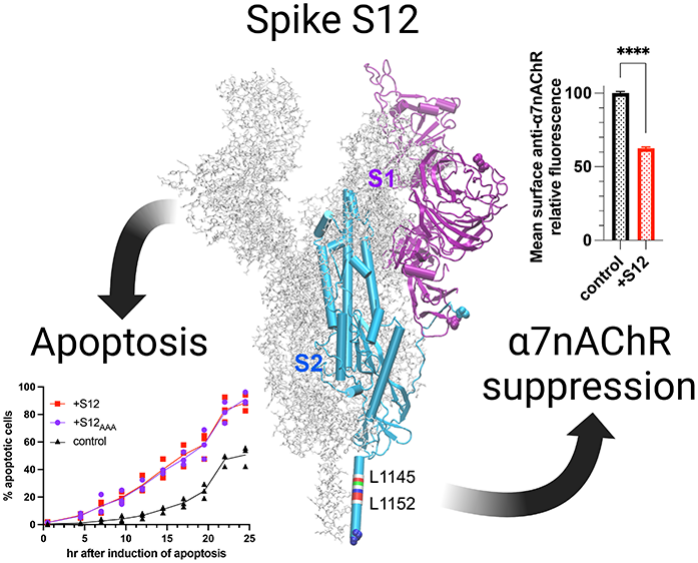

A deficiency of the functional α7 nicotinic acetylcholine receptor (α7nAChR) impairs neuronal and immune systems. The SARS-CoV-2 spike protein (S12) facilitates virus cell entry during COVID-19 infection and can also independently disrupt cellular functions. Here, we found that S12 expression significantly downregulated surface expression of α7nAChR in mammalian cells. A helical segment of S12 (L1145-L1152) in the spike neck was identified to be responsible for the downregulation of α7nAChR, as the mutant S12_AAA_ (L1145A-F1148A-L1152A) had minimal effects on surface α7nAChR expression. This S12 segment is homologous to the α7nAChR intracellular helical motif known for binding chaperone proteins RIC3 and Bcl-2 to promote α7nAChR surface expression. Competition from S12 for binding these proteins likely underlies suppression of surface α7nAChR. Considering the critical roles of α7nAChR in cellular functions, these findings provide a new perspective for improving mRNA vaccines and developing treatment options for certain symptoms related to long COVID.

## Introduction

SARS-CoV-2 infects human cells through its spike protein that comprises two major domains, S1 and S2. S1 is responsible for recognition and binding to the angiotensin-converting enzyme 2 (ACE2) receptor on the surface of target cells, while S2 mediates fusion of viral and host cell membranes. The spike protein facilitates virus cell entry that leads to an acute infection known as COVID-19.^1−4^ A subpopulation of patients experiences long-term sequelae from the infection, known as long COVID, which is characterized by a wide range of health issues,^1,5−7^ including brain fog and other neurological and psychiatric problems.^1,5,6,8,9^ The precise cause of long COVID is yet to be determined, but it is unlikely through a single mechanism. Direct action of the spike protein, S12, has been considered as a potential cause for some of the detrimental effects of SARS-CoV-2.^10^ Immunoreactive S12, suspected to contribute to cardiovascular disease independent of viral infection, was found circulating in the blood of COVID-19 patients.^11^ S12 can act alone or in conjunction with other mediators on target cells, stimulate different cell types, damage the integrity of the blood–brain barrier, and contribute to the pathogenesis of long COVID.^10,12−15^ These findings undoubtedly implicate S12 as an inducer of cellular dysfunction.

The α7 nicotinic acetylcholine receptor (α7nAChR) was linked to COVID-19 pathophysiology at the early stage of the pandemic.^16^ α7nAChR forms homopentameric ligand gated ion channels that mediate synaptic transmission in the central and peripheral nervous systems.^17,18^ It is involved in cognitive function, mental health, and neurodegenerative diseases.^19^ α7nAChR is also a major player in the cholinergic anti-inflammatory pathway,^20,21^ which attenuates proinflammatory cytokine production and minimizes tissue and organ injury during inflammation. The α7nAChR agonists, such as nicotine, are essential for initiating the cholinergic anti-inflammatory pathway and effective in reducing macrophage cytokine production and inflammation.^22,23^ Therapeutic implications of cholinergic signaling in acute and chronic pathology, including a therapeutic avenue for treating COVID-19, have been supported by human data and animal studies.^24−26^ α7nAChR is widely expressed across the human body in both neuronal and non-neuronal cells.^19,24,27−30^ In the brain, it is expressed on both pre- and postsynaptic membranes and particularly in regions implicated in cognitive function, such as the hippocampus and cortex.^31,32^ α7nAChR is also expressed in immune cells, such as macrophages, that form the basis for some of the known α7nAChR-mediated anti-inflammatory effects.^27^ A deficiency of functional α7nAChR is implicated in neuropsychic diseases and disrupts the cholinergic anti-inflammatory pathway.^30,33−38^

In this study, we discovered that the SARS CoV-2 spike protein ectodomain (S12) can significantly suppress expression of α7nAChR in mammalian cells. The suppression has a much more profound impact on surface α7nAChR than that in the intracellular stores, implying that S12 mainly affects receptor trafficking. The suppression effect results from S12 coexpression with α7nAChR instead of the S12 presence in extracellular milieu. Additionally, we have identified a segment in S2 that is homologous to the hydrophobic helical motif in the α7nAChR intracellular domain, which is responsible for binding the receptor chaperone proteins, such as resistance to inhibitor of cholinesterase-3 (RIC3)^39^ and antiapoptotic Bcl-2 family proteins.^40^ Site-directed mutagenesis of the S2 segment abolishes the profound suppression of surface α7nAChR and S12 can pull RIC3 down, suggesting that S12 competition for binding to chaperone proteins is likely an underlying mechanism leading to suppression of surface α7nAChR when S12 is coexpressed. These findings provide a new perspective for understanding certain symptoms of COVID 19 and long COVID,^5−7,41,42^ and for aiding in the design of potential new treatments for post-COVID syndromes.

## Results and Discussion

### Expression of S12 in PC12 Cells Suppresses Native Surface α7nAChR

**Figure 1:**
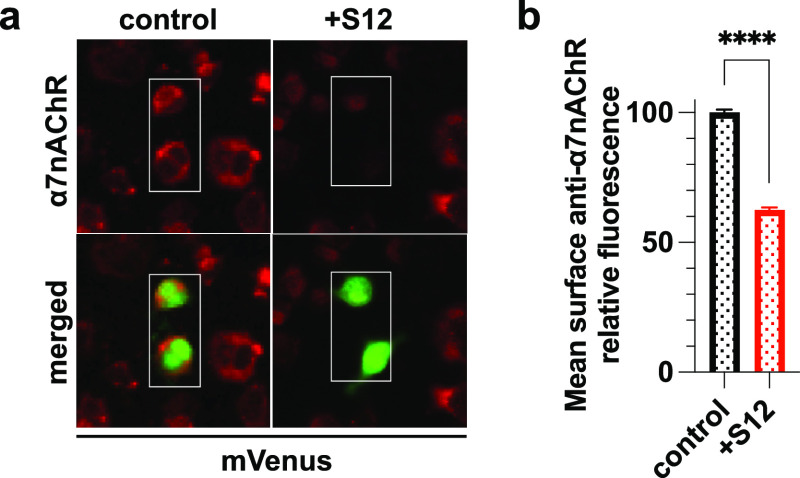
S12 expression downregulates native surface α7nAChR in PC12 cells. (a) Immunofluorescent staining of PC12 cells transfected with cDNA encoding mVenus (control) or co-transfected with cDNAs encoding mVenus and S12 (+S12). Surface expression of α7nAChR was detected using anti-α7nAChR antibody (1:200, Invitrogen PA5-115651) in nonpermeabilized cells. Boxed areas indicate representative transfected cells (green), for which the α7nAChR labeling intensity (red) was measured. (b) Normalized mean relative intensities of anti-α7nAChR staining showing ∼37% reduction of α7nAChR labeling in the +S12 group compared to the control. Data collection was performed ∼36 h after transfections. Data for each group were collected from three independent experiments with a total *n* = 4689 (control) and *n* = 3293 (+S12) cells. Data are the mean ± SEM; *p* ≤ 0.0001 (****) from two-tailed unpaired *t* test.

PC12 cells contain native α7nAChR and are well-established and commonly used for studies of neuroinflammation.^43^ We measured surface expression of α7nAChR in intact nonpermeabilized PC12 cells using immunocytochemistry with anti-α7nAChR primary antibody and an Alexa Fluor 594 conjugated secondary antibody (Figure 1a). Transfected cells were identified by the expression of a green fluorescent protein (mVenus), either without (control) or with S12 (+S12). The mean intensity of the α7nAChR staining for each transfected cell was quantified and normalized by the mean intensity for the control group. The PC12 cells co-transfected with plasmids expressing mVenus+S12 showed ∼37% reduction in cell surface α7nAChR relative to those in the control group (Figure 1b).

### Coexpression of S12 with α7nAChR Suppresses Surface Expression of α7nAChR in HEK293 Cells

**Figure 2:**
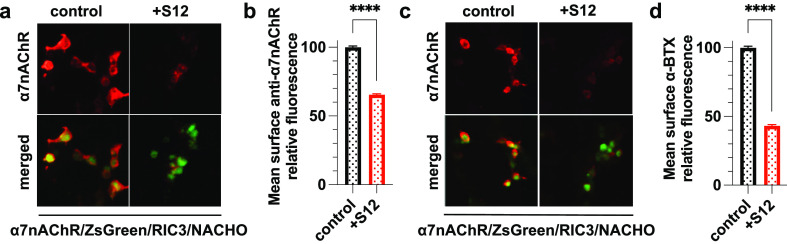
Coexpression of S12 downregulates surface α7nAChR in HEK293T/17 cells. (a) Immunofluorescent staining of nonpermeabilized HEK293T/17 cells co-transfected with cDNAs encoding α7nAChR, ZsGreen, RIC3, NACHO without (control) or with S12 (+S12). Surface expression of α7nAChR (red) was detected using anti-α7nAChR antibody (1:200, Invitrogen PA5-115651). (b) Relative mean intensities of anti-α7nAChR staining show ∼35% reduction of surface α7nAChR in the +S12 group compared to the control. Data for each group were from three independent experiments with a total *n* = 9010 (control) and *n* = 7636 (+S12) cells. (c) Fluorescent staining of nonpermeabilized HEK293T/17 cells co-transfected with the same cDNAs as described in (a). Surface expression of functional α7nAChR (red) was detected using live cell labeling with 1 μg/mL α-bungarotoxin-Alexa Fluor 594 conjugate (α-BTX, red). (d) Relative mean intensities of α-BTX staining show ∼57% reduction of functional α7nAChR in the +S12 group compared to the control. Data collection was performed ∼36 h after transfections. Data for each group were collected from two independent experiments with a total *n* = 3384 (control) and *n* = 1587 (+S12) cells. All data are the mean ± SEM; *p* ≤ 0.0001 (****) from two-tailed unpaired *t* test.

HEK293 cells show negligible α7nAChR expression unless in the presence of protein chaperones to promote α7nAChR trafficking and biogenesis.^40,44−46^ This feature enables investigations into S12 effects on recombinant α7nAChR expression. We transfected HEK293 cells with plasmid constructs expressing α7nAChR with or without coexpression of S12 in addition to cDNAs encoding the green fluorescent protein ZsGreen and the chaperones (RIC3 and NACHO) marked in Figure 2. The transfected cells showed robust surface expression of α7nAChR as measured by labeling with anti-α7nAChR antibody (Figure 2a) or with an Alexa Fluor 594 conjugate of α-bungarotoxin (αBTX) (Figure 2c), which is an α7nAChR-selective antagonist derived from snake venom and its binding indicates functional α7nAChR.^17^ Compared to the control group, coexpression of S12 in HEK293 cells reduced surface α7nAChRs by ∼35% (Figure 2b), which is similar to the observed ∼37% reduction in PC12 cells (Figure 1b). Functional α7nAChR capable of binding αBTX was reduced by ∼57% due to S12 coexpression in HEK93 cells (Figure 2d).

### S12 Coexpression Did Not Affect Surface Expression of α4β2nAChR

**Figure 3:**
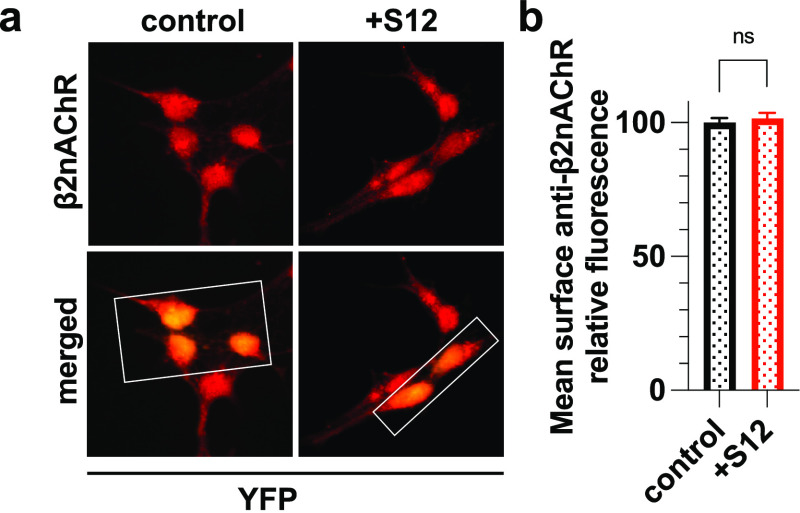
Coexpression of S12 with α4β2nAChR did not affect surface α4β2 expression. (a) Immunofluorescent staining of nonpermeabilized HEK293 cells stably expressing α4β2nAChR that were transiently transfected with cDNA encoding YFP (control) or also with S12 (+S12). Surface β2nAChR was detected using anti-β2nAChR antibody (1:200, Invitrogen PA5-77508). Boxed areas highlight representative transfected cells (yellow) for which the β2nAChR labeling intensity (red) was measured. (b) Quantified relative mean intensities of anti-β2nAChR staining show no significant difference in surface β2nAChR labeling between the +S12 and control groups. Data collection was performed ∼36 h after transfections. Data for each group were from two independent experiments with a total number of cells *n* = 1537 (control) and *n* = 1289 (+S12). Data are the mean ± SEM; *p* > 0.05 (ns) from two-tailed unpaired *t* test.

Does S12 expression also downregulate other subtypes of nAChRs? To answer this question, we examined surface expression of α4β2nAChR, a major subtype of nAChRs in the brain.^32^ In contrast to what we observed on α7nAChRs, S12 coexpression had no impact on cell surface expression of α4β2nAChR (Figure 3). The distinctly different responses from α7nAChR and α4β2nAChR suggest that S12 influence on surface receptor expression may depend on receptor subtypes. However, future studies with additional subtypes of nAChRs are required for a definite answer as to whether S12-caused suppression of surface receptor expression is limited only to α7nAChR.

### Suppression of Functional α7nAChR on Cell Surface Does Not Result from S12 in Extracellular Media

**Figure 4:**
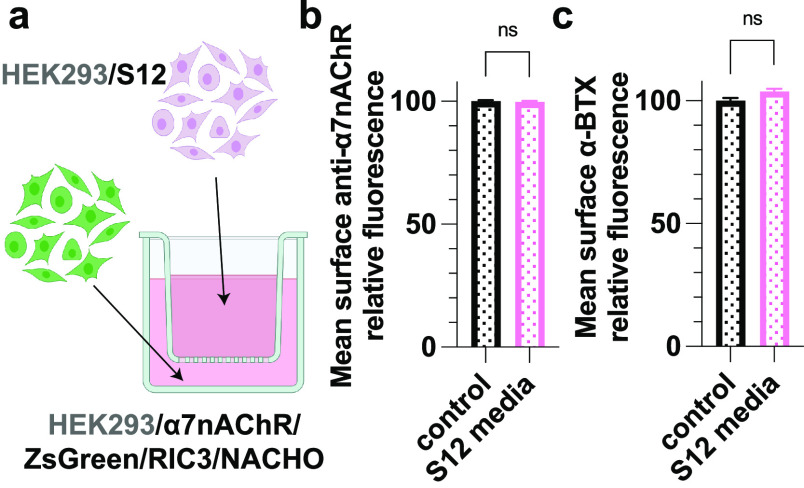
S12 in the media does not suppress surface α7nAChR expression. (a) Schematic presentation of coculture differently transfected cells that share media but are physical separated by a porous (0.4 μm) membrane. Modified from Biorender.com. (b) Relative mean intensities of anti-α7nAChR labeling of nonpermeabilized HEK293T/17 cells transiently co-transfected with cDNAs encoding α7nAChR, ZsGreen, RIC3, and NACHO. Media was shared with cells in the well inserts transiently transfected with cDNA encoding mVenus (control) or co-transfected with mVenus +S12 (S12 media) for 5 days. The presence of secreted S12 in the S12 media was confirmed by Western blot (Figure S2b). No significant difference was observed between groups. Data for each group were from two independent experiments with a total number of cells *n* = 21 066 (control) and *n* = 20 677 (S12 media). (c) Relative mean intensities of α-bungarotoxin (α-BTX) labeling of functional α7nAChR from the same groups as in **(**b) with a total of *n* = 5592 (control) and *n* = 4871 (S12 media) cells. All data are the mean ± SEM; *p* > 0.05 (ns) from two-tailed unpaired *t* test.

The spike protein ectodomain S12 is largely soluble. It can be secreted into cell culture media and even purified from culture supernatant.^47^ Could surface α7nAChR be suppressed through an extracellular action of S12? We found that expression of surface α7nAChR and its binding to αBTX were unaffected by external S12, either through adding ∼400 nM of the purified HexaPro trimer (a stabilized S12 variant) to the culture media (Figure S1) or through replacing the culture media of α7nAChR with the conditioned media from cells expressing S12 (Figure S2). In the latter case, the secreted S12 from HEK293 cells transfected with cDNA encoding S12 in the conditioned media was confirmed by Western blot (Figure S2b). To further evaluate the possibility of an extracellular S12 action, we utilized the Transwell (Corning) that physically separates cells expressing α7nAChR from those expressing S12 but allows the secreted S12 to pass the porous membrane (0.4 μm) of the Transwell to bathe cells expressing α7nAChR (Figure 4a). Again, surface α7nAChR expression (Figure 4b) and αBTX binding (Figure 4c) were unaffected even after being cultured in Transwell for up to 5 days. These results suggest that S12 in extracellular media does not play a role in downregulating surface α7nAChR and disrupting αBTX binding to α7nAChR.

### S12 Expression Has Almost No Effect on Intracellular α7nAChR Stores

**Figure 5:**
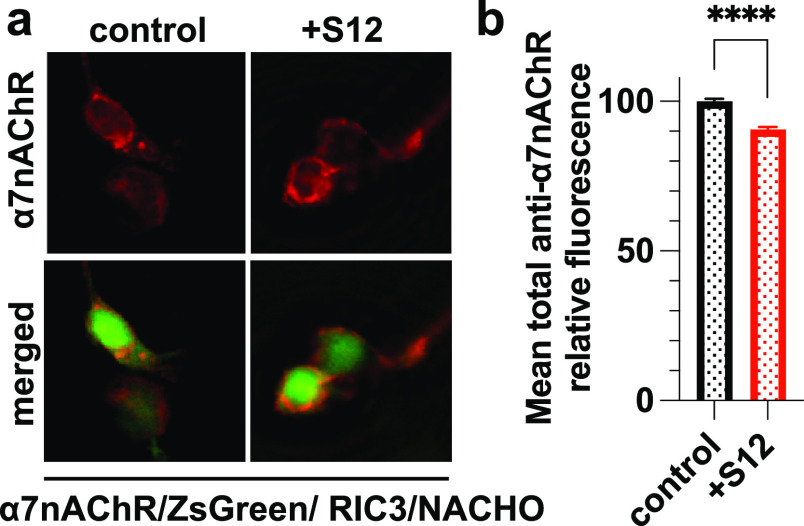
Coexpression of S12 has a small but significant effect on total α7nAChR expression. (a) Immunofluorescent staining of permeabilized HEK293T/17 cells co-transfected with cDNAs encoding α7nAChR, ZsGreen, RIC3, NACHO without (control) or with S12 cDNA (+S12). Total expression of α7nAChR (red) was detected using anti-α7nAChR antibody (1:200, Invitrogen PA5-115651) after detergent permeabilization. (b) Relative mean intensities of anti-α7nAChR staining show ∼9% reduction of α7nAChR labeling in the +S12 group compared to the control. Data collection was performed ∼36 h after transfections. Data for each group were from two independent experiments with a total *n* = 8867 (control) and *n* = 6655 (+S12) cells. All data are presented as the mean ± SEM; *p* ≤ 0.0001(****) from two-tailed unpaired *t* test.

Downregulation of surface α7nAChR could result from a change in receptor trafficking or a decrease of intracellular α7nAChR stores. To differentiate these possibilities, we used permeabilized cells to measure a total reduction of α7nAChR expression due to S12 coexpression in HEK293 cells. We performed immunocytochemistry with anti-α7nAChR antibody after cell permeabilization (Figure 5a). In contrast to the ∼35% reduction of surface α7nAChRs measured in nonpermeabilized cells (Figure 2b), only a ∼9% reduction of total α7nAChRs was observed in permeabilized cells (Figure 5b). Given the known distributions of surface (34 ± 3%) and intracellular (66 ± 3%) receptors,^48^ our results suggest that coexpression of S12 has almost no impact on the intracellular α7nAChR pool.

### A S2 Helical Segment in the Spike Neck Homologous to the Chaperone-Binding Motif of α7nAChR Is Required to Downregulate Surface α7nAChR

**Figure 6:**
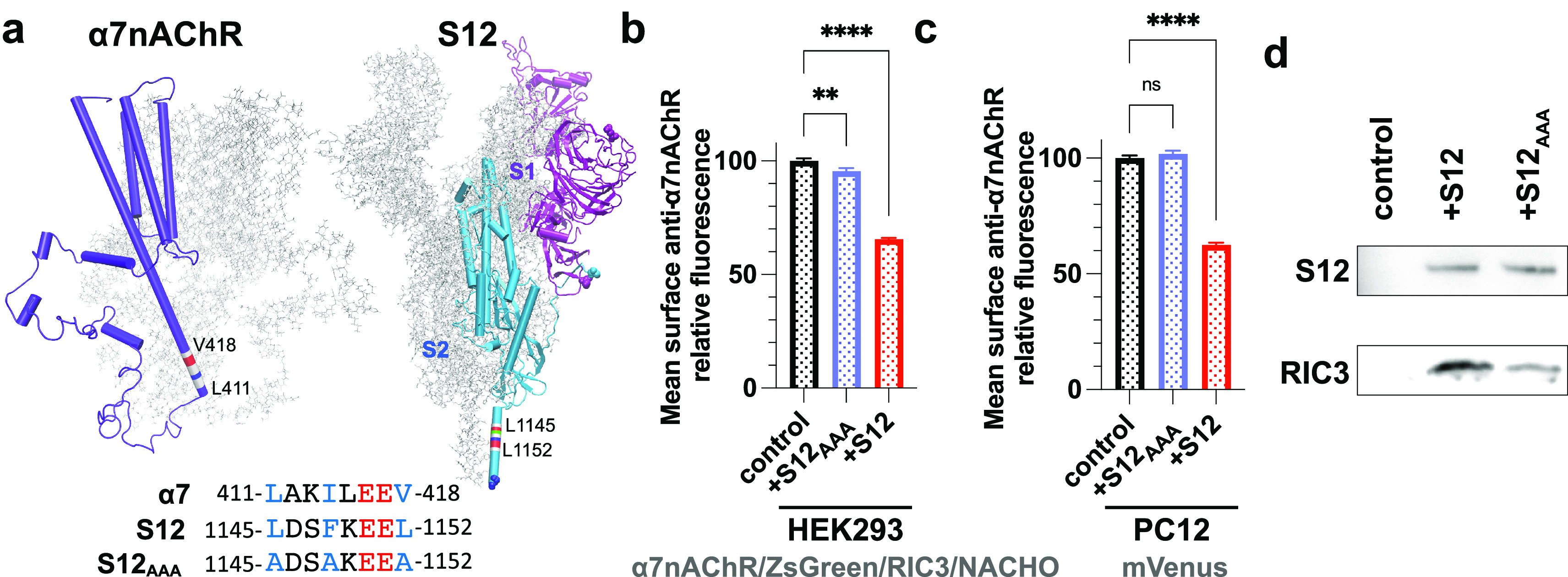
A helical segment of S12 (1145L-1152L) homologous to the intracellular chaperone-binding motif of α7nAChR (L411-V418) is primarily responsible for the S12-induced downregulation of surface α7nAChR. (a) Structures of α7nAChR transmembrane and intracellular domains (PDB code 7RPM) and S12 (PDB code 7VXF) showing the helical motif known for binding α7nAChR chaperones. Sequence alignments of the helical segments highlight three hydrophobic residues (blue) critical for binding α7nAChR chaperones and their mutations in S12_AAA_. (b) Relative mean intensities of anti-α7nAChR immunofluorescent staining of nonpermeabilized HEK293 cells showing that relative to the control group (α7nAChR/ZsGreen/RIC3/NACHO), coexpressing S12_AAA_ (+S12_AAA_) caused only ∼5% reduction of surface α7nAChR in contrast to ∼35% reduction by S12 coexpression (+S12, adapted from Figure 2). Data for each group were from two independent experiments with a total number of cells *n* = 8594 (control) and *n* = 4903 (+S12_AAA_). (c) Relative mean intensities of anti-α7nAChR immunofluorescent staining of nonpermeabilized PC12 cells transiently transfected with cDNA of mVenus alone (control) or +S12_AAA_. In contrast to ∼37% reduction by S12 coexpression (red, adapted from Figure 1), +S12_AAA_ coexpression (blue) produced no significant difference in the staining intensity compared to the control. (d) RIC3 pulldown by S12 or S12_AAA_ from lysates of HEK293 cells coexpressing RIC3 and S12 or S12_AAA_. The His-tags on the S12 constructs were used for pulldowns using NiNTA resin. The bound proteins were probed by anti-RIC3 (1:100) and anti-SARS-CoV-2 spike protein (1:250). RIC3 binding to S12_AAA_ was much reduced compared to S12. RIC3 was not detected in the absence of S12. Data collection was performed ∼36 h after transfections. Data for each group in (b) and (c) were from three independent experiments with a total number of cells *n* = 7853 (control) and *n* = 5614 (+S12_AAA_). Data are the mean ± SEM; *p* values are from one-way ANOVA with Dunnett’s multiple comparisons; *p* > 0.05 (ns), *p* ≤ 0.01 (**), *p* ≤ 0.0001 (****).

Chaperone proteins of α7nAChR, including neuronal-specific transmembrane protein 35a (NACHO),^45^ RIC3,^49^ and antiapoptotic B-cell lymphoma 2 (Bcl-2) proteins,^40^ play a critical role in assembly, trafficking, and ultimately surface expression of α7nAChR. NACHO exerts its action on α7nAChR expression without directly interacting with α7nAChR,^50^ but RIC3 and Bcl-2 interact directly with an intracellular helical segment (L411-V418) of α7nAChR (Figure 6a). The α7nAChR mutations, by replacing large-size hydrophobic residues with alanine in this segment (L411A-I414A-V418A), reduced surface expression of the receptor due to poor interactions of the mutant with antiapoptotic Bcl-2 proteins.^40^ The same helical segment was also found responsible for α7nAChR expression promoted by the chaperone protein RIC3, as mutations of L411A or V418A abolished or largely weakened RIC3-mediated expression of α7nAChR.^39^ In contrast, mutations on hydrophilic residues, such as K413A, had little effect.^39^ With this knowledge from previous studies, we questioned if S12 contains a similar helical motif that may compete for binding the chaperone proteins and thereby weaken their effects on α7nAChR surface expression. After searching sequence homologies (see the method section), we identified a homologous helical segment (L1145-L1152) (Figure 6a) in the spike neck,^51^ a region in the S2 subunit between spike head and stalk. We mutated this S12 segment (named S12_AAA_) by replacing the large-size hydrophobic residues with alanine (L1145A-F1148A-L1152A) (Figure 6a). As we predicted, S12_AAA_ coexpression imposed only ∼5% reduction of surface α7nAChR, much smaller than that caused by S12 coexpression (Figure 6b), even though S12_AAA_ expression level is ∼9% higher than S12 expression in HEK cells (Figure S3). In PC12 cells, expression of S12_AAA_ did not downregulate native α7nAChR expression on the cell surface (Figure 6c). We further performed pulldown experiments on HEK293 cells coexpressing RIC3 and S12 or S12_AAA_ to support the hypothesis that S12 competes for binding chaperone proteins and thereby weaken their effects on α7nAChR surface expression. RIC3 pulldown by S12 (Figure 6d) supports the hypothesized S12 binding to RIC3. The pulldown experiment also confirms that the helical segment (L1145-L1152) of S12 is involved in the binding. Relative to S12, S12_AAA_ shows much less RIC3 binding under the same experimental condition in the same assay (Figure 6d). A ∼67% decrease in RIC3 binding to S12_AAA_ vs S12 is observed based on the integrated intensity using ImageJ.^52^ Taken together, these results suggest that the L1145-L1152 segment in the spike neck downregulates surface α7nAChR expression. The sequence homology between this S12 segment and the chaperone-binding motif of α7nAChR enables S12 to compete with α7nAChR for binding chaperones and consequently leads to a decreased surface expression of α7nAChR.

### S12 and S12_AAA_ Accelerate Apoptosis

**Figure 7:**
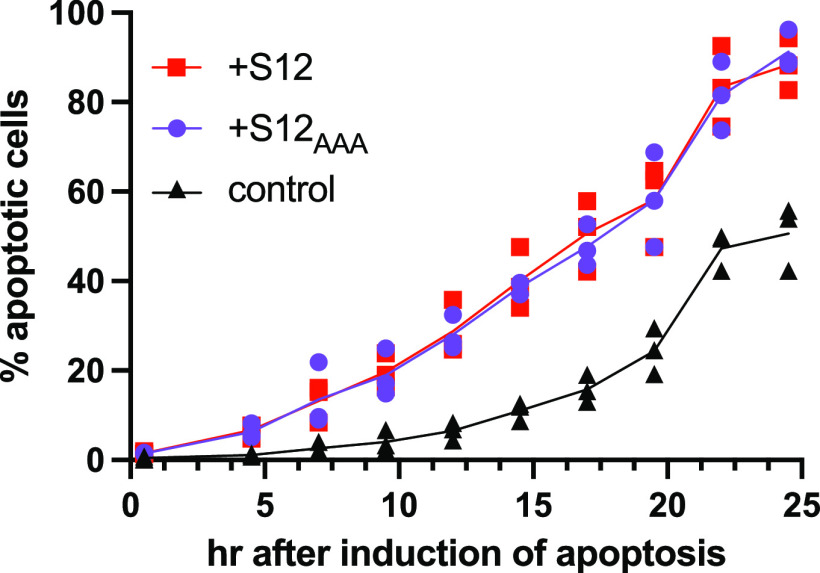
Both S12 and S12_AAA_ accelerate apoptosis. Time course of apoptosis in differentiated PC12 cells transfected with cDNAs encoding mCerulean (control, black triangles) or mCerulean plus either S12 (red squares) or S12_AAA_ (purple circles). Apoptosis was induced by serum deprivation and 1 μM staurosporine and measured as the percentage of transfected cells with activated caspase-3/7 activity. The measurements were performed ∼4 days after transfections. Data for each group were from three independent experiments with a total number of cells *n* = 3076 (control), *n* = 2170 (+S12_AAA_), and *n* = 3085 (+S12).

In addition to evaluating suppression of surface α7nAChR, we also tested if S12 expression could accelerate cell apoptosis. Using differentiated PC12 cells, we measured caspase-3/7-dependent apoptosis^53^ in cells expressing S12 or S12_AAA_ relative to the control. The rate of apoptosis was monitored over 24 h by measuring caspase-3/7 activity after induction by serum deprivation^54^ and 1 μM staurosporine.^55^ S12 expression significantly increased the rate of apoptosis relative to the control (Figure 7). Interestingly, S12_AAA_ expression led to a similar result (Figure 7). Thus, the primary mechanism of S12 acceleration of apoptosis in PC12 cells is not related to the L1145-L1152 segment and not dependent on S12 inhibition of α7nAChR surface expression.

A major discovery from the current study is the downregulation of surface α7nAChR by the spike protein ectodomain S12. The extent of the downregulation is profound, with more than one-third of surface α7nAChR and more than half of the functional α7nAChR (Figures 1 and 2). The α7nAChR downregulation by S12 may be ubiquitous across different cell types because it has happened in both neuronal PC12 cells and HEK293 cells expressing native and recombinant α7nAChR (Figures 1 and 2), respectively. It is known that α7nAChR is expressed in various cells and has functions in both neuronal and immune systems.^19,24,27−30^ A significant depletion of functional α7nAChR, such as that caused by coexpression of S12 (Figure 2), would have a negative impact on neuronal circuitry and regulation of anti-inflammatory signaling.^30,33−38^ Deficiency of functional α7nAChR in the hippocampus and other brain regions is associated with neuropsychiatric disorders with numerous deleterious symptoms, including cognitive impairments and sensory processing deficits.^36−38^ Enhancing α7nAChR function, on the other hand, can improve cognitive performance, attention, and memory.^30^ α7nAChR is a key player in the cholinergic anti-inflammatory pathway.^27^ Its presence in immune cells is critical for α7nAChR-mediated anti-inflammatory signaling and neuroprotection.^56^ Thus, a significant reduction of α7nAChR can potentially impair not only cognitive performance and sensory processing^36−38^ but also normal immune response to inflammation.^56^ The discovered S12 effect on the downregulation of surface α7nAChR provides a new perspective for understanding certain symptoms of COVID-19, particularly cognitive and immunity-related symptoms of long COVID.^5−7,41,42^

Another major discovery from the current study is the underlying mechanism of how S12 downregulates surface α7nAChR. It is intriguing to learn that a helical segment (L1145-L1152) in the spike neck is homologous with the α7nAChR helical segment (L411-V418) responsible for binding chaperones for receptor assembly and trafficking. Eliminating the possibility for S12 binding α7nAChR chaperones (RIC-3 and antiapoptotic Bcl-2 proteins) in S12_AAA_ restored surface α7nAChR close to normal (Figure 6b,c). The result supports a competition mechanism for downregulating surface α7nAChR by S12. This competition mechanism may also explain why S12 coexpression did not affect the surface population of α4β2nAChRs (Figure 3). Both α4 and β2 miss a bulky hydrophobic residue critical for binding the chaperones found in α7nAChR (Figure S4).^40^ It is worth mentioning that the L1145-L1152 segment is conserved in all variants of SARS-CoV-2. The predominant SARS-CoV-2 mRNA vaccines encode the full-length spike protein,^57,58^ including the L1145-L1152 segment. For new versions of SARS-CoV-2 mRNA-based vaccines, one should consider mutating the L1145-L1152 segment, as we did for S12_AAA_, to prevent potential adverse effects.

A potential contribution of α7nAChR to COVID-19 pathophysiology was proposed based on the sequence homology found in S12 and snake venom neurotoxins, including α-BTX,^16^ which are potent competitive antagonists of α7nAChR. The predicted interactions were demonstrated by computational studies between α7nAChR and a S12 peptide (Y674-R685) homologous to α-BTX.^59^ A recent report showed experimental evidence supporting the S12 peptide interactions with α7nAChR and functional impact of the interactions on α7nAChR.^60^ Isolated S1 (at a concentration as low as 1 nM) was recently found to inhibit currents of *Xenopus* oocytes expressing α7nAChR.^61^ Thus far, however, no experimental result shows direct interactions between the intact S12 trimer and α7nAChR. In the current study, we did not observe a significant change in the α-BTX staining of α7nAChR when S12 was present in cell culture media (Figure S1). Steric hindrance of its bulky trimer may have prevented S12 from access to a potential binding site of α7nAChR, or S12 simply could not compete with high-affinity α-BTX binding. Nevertheless, a more comprehensive functional study is required to define a role of an intact S12 trimer in the channel function of α7nAChR.

A recent study showed that extracellular application of recombinant SARS-CoV-2 spike protein or infection with SARS-CoV-2 spike pseudovirions promoted cell apoptosis through upregulating the intracellular reactive oxygen species (ROS), though the mechanism of the spike protein upregulation of ROS remains unknown.^62^ Our apoptosis data show that S12 or S12_AAA_ expression in PC12 cells accelerated apoptosis compared to the control group (Figure 7). In these experiments the spike protein was both expressed intracellularly and secreted to the cell media so that the contributions of external and internal S12 to apoptosis could not be distinguished. Similar acceleration rates of apoptosis by S12 and S12_AAA_ (Figure 7) suggest that the L1145-L1152 segment and the resulting suppression of surface α7nAChR expression are not dominant factors in the apoptotic action of S12. Nevertheless, exact mechanisms of S12-induced acceleration of apoptosis and other adverse effects^63^ warrant further investigations in the future.

How long can S12 remain in the human body to potentially contribute to postinfection symptoms? Elevated levels of immunoreactive S12 were detected in the serum of COVID-19 patients aged 46 or older between 5 and 10 days from the onset of COVID-19 symptoms.^11^ According to the United States Center for Disease Control and Prevention (https://www.cdc.gov/coronavirus/2019-ncov/hcp/duration-isolation.html), patients who have recovered from COVID-19 can have detectable SARS-CoV-2 RNA in upper respiratory specimens for up to three months after onset of illness. Detection of subgenomic SARS-CoV-2 RNA has been reported in moderately or severely immunocompromised patients up to >140 days after a positive SARS-CoV-2 test result.^64,65^ Recently, the presence of an active persistent SARS-CoV-2 viral reservoir was suggested based on detected SARS-CoV-2 spike antigen in postacute sequelae of COVID-19 patients up to 12 months postdiagnosis.^66^ If the viral reservoir and persistent spike replication are confirmed, S12-induced surface α7nAChR suppression found in the current study can likely impose prolonged negative impact to neuronal and immune processes requiring functional α7nAChRs. Any cells expressing spike protein are likely to have a depressed α7nAChR response. The effect may vary depending on cell types. It requires further investigation on more cell types before one can conclude how much a viral reservoir leads to α7nAChR suppression and consequently contribute to the various symptoms of long COVID. Nevertheless, the circumstances resulting in persistently detectable SARS-CoV-2 RNA, spike, or spike antigen and how they contribute to long-COVID have yet to be determined. Their persistent presence raises a significant concern for patients recovered from COVID-19 but chronically immunocompromised. The potential downregulation of functional α7nAChR due to S12 coexpression as found in this study, and impairment of neuronal and immune functions associated with α7nAChR deficiency, should be considered in the future for formulating treatment options for these patients.

## Materials and Methods

### Cell Models

PC12 cells (male, rat adrenal gland pheochromocytoma, RRID: CVCL_0481) were obtained from the ATCC (ATCC catalog no. CRL-1721) and maintained at 37 °C and 5% CO_2_ in a humidified incubator. Growth medium was F12K (Thermo Fisher catalog no. 21127–030) supplemented with 10% horse serum (Thermo Fisher catalog no. 26050088), 5% fetal bovine serum (Thermo Fisher catalog no. 16140071), and 1% penicillin/streptomycin (Cytiva catalog no. SV30010) on collagen-coated plates or glass coverslips. Cells were maintained at low passage number from the source but were not otherwise authenticated during these experiments. PC12 cells were transfected using the Transporter 5 transfection reagent (PolySciences catalog no. 26008-5) following the manufacturer’s instructions. Transfected cells comprised three experimental groups: control, transfected only with mVenus C1 (RRID: Addgene_27794); +S12, co-transfected at a 1:1 ratio with mVenus C1 and pαH-S-RRAR (RRID: Addgene_164569); and +S12_AAA_, co-transfected at a 1:1 ratio with mVenus C1 and pαH-S-AAA. For measuring apoptosis in differentiated PC12 cells, three groups include control, transfected only with mCerulean C1 (RRID: Addgene_27796); +S12, co-transfected at a 1:1 ratio with mCerulean C1 and pαH-S-RRAR; and +S12_AAA_, co-transfected at a 1:1 ratio with mCerulean C1 and pαH-S-AAA. Cells were differentiated in F12K media supplemented with 1% horse serum, 0.5% fetal bovine serum, and 100 ng/mL neural growth factor (Thermo Fisher catalog no. 13257-019) for 3 to 5 days. Cells were maintained at low passage number from the source but were not otherwise authenticated during these experiments.

HEK293T/17 cells (female, human fetal kidney, RRID: CVCL_1926) were obtained from the ATCC (ATCC catalog no. CRL-11268) and maintained at 37 °C and 5% CO_2_ in a humidified incubator. Growth medium was DMEM, high glucose, pyruvate (Gibco catalog no. 11995-065) supplemented with 10% fetal bovine serum (Thermo Fisher catalog no. 16140071), and 1% penicillin/streptomycin (Cytiva catalog no. SV30010). Cells were maintained at low passage number from the source but were not otherwise authenticated during these experiments. For imaging studies, cells were grown on collagen-coated 24 well plates or glass coverslips. HEK293T/17 cells were transfected using the DOTAP liposomal transfection reagent (Roche catalog no. 11202375001) following the manufacturer’s instructions. Transfected cells comprised three experimental groups: control, co-transfected at a 3:2:1 ratio with pLenti6-α7nAChR-ZsG, pLX304-RIC3 (DNASU catalog no. HsCD00438164), and pCMV3-TMEM35 (NACHO, Sino Biological catalog no. HG27483-UT); +S12, co-transfected at a 3:2:1:3 ratio with pLenti6-α7nAChR-ZsG, pLX304-RIC3, pCMV3-TMEM35, and pαH-S-RRAR; and +S12_AAA_, co-transfected at a 3:2:1:3 ratio with pLenti6-α7nAChR-ZsG, pLX304-RIC3, pCMV3-TMEM35 (NACHO), and pαH-S-AAA (RRID: Addgene_164569).

KXα4β2 stably transfected HEK293 cells^67^ were maintained and transfected as described above for HEK293T/17, except 0.7 mg/mL G418 (Sigma catalog no. G8168) was added to maintain selection of α4β2nAChR. Cells were maintained at low passage number from the source but were not otherwise authenticated during these experiments. Transfected cells comprised two experimental groups: control, transfected with pcDNA3-YFP (RRID: Addgene_13033); and +S12, co-transfected at a 1:1 ratio with pcDNA3-YFP and pαH-S-RRAR.

### Plasmids

Human α7nAChR was subcloned from pMXT-α7AChR, a gift from J. Lindstrom^68^ with primers a7_fwd (tctagaggatcgtcgccaccatgcgctgctcgccggga) and a7_rev (ctagactcgatgatcagttactacgcaaagtctttggacacggcc) to the mammalian dual expression vector pLenti6-CMV-RFPn-CMV-ZsG (a gift from Bing Wang, University of Pittsburgh) replacing RFPn using overlapping PCR^69^ with primers v_fwd (taactgatcatcgagtctagaggg) and v_rev (ggtggcgacgatcctctaga) to create pLenti6-α7nAChR-ZsG. pLX304-RIC3 was obtained from DNASU (clone HsCD00438164). pCMV3-TMEM35 was purchased from Sino Biological (catalog no. HG27483-UT). SARS-CoV-2 S HexaPro (stabilized S12, RRID: Addgene_154754^70^), pαH-S-RRAR (wild-type S12, RRID: Addgene_164569^47^), mVenus C1(RRID: Addgene_27794^71^), and pcDNA3-YFP(RRID: Addgene_13033, Doug Golenbock) were obtained from Addgene. pαH-S-AAA was constructed from pαH-S-RRAR by PCR mutagenesis using the following primers S12AAA_begin_fwd (tgtgctgaacgatatcctgtctagactggacaaggtgg) S12AAA_begin_rev (ggcctcttccttggcagagtcggcctcaggttgcagagggtc) S12AAA_end_fwd (gccgactctgccaaggaagaggccgacaagtactttaaaaaccacaccagcc) S12AAA_end_rev (ctatgaccatgattacgccaagcttgggctgcaggtcg).

### Immunocytochemistry and Fluorescent Labeling

Transfected cells were expressed for ∼36 h and washed with media or Dulbecco’s phosphate buffered saline (DPBS) before being labeled. For bungarotoxin live labeling, α-bungarotoxin Alexa Fluor 594 conjugate (Invitrogen catalog no. B13423) was added at 5 μg/mL media and incubated at 37 °C for 30 min, then washed three times with DPBS and fixed for imaging with 4% paraformaldehyde (PFA). For immunocytochemistry, the washed cells were fixed in 4% PFA and then treated with R&D acidic antigen retrieval reagent (R&D catalog no. CTS014) for 5 min at 90 °C. The cells were incubated with blocking buffer (10 mg/mL bovine serum albumin and 5% goat serum (Sigma catalog no. G9023) in DPBS) for 1 h and incubated with primary antibody in blocking buffer at 4 °C overnight. After washing three times in DPBS, the cells were incubated with 1:850 Alexa Fluor-594 conjugated secondary anti-rabbit antibody (Thermo Fisher catalog no. A-11012; RRID: AB_2534079) in blocking buffer for 2 h at room temperature. After washing 3× in DPBS, the cells were fixed and ready for imaging. Primary antibodies were anti-α7nAChR polyclonal (RRID: AB_2900286), SARS-CoV-2 spike protein (RBD) polyclonal (RRID: AB_2890581), or CHRNB2 polyclonal (RRID: AB_2735656) as indicated in the figure legends. Where indicated, cells were permeabilized by including 0.05% Tween 20 (Fisher catalog no. BP337-100) in the antigen retrieval reagent and 0.2% Triton X-100 (Sigma catalog no. T8787-100ML) in the blocking buffer and antibody incubations. All fluorescence images were collected using uniform exposure settings with an Olympus IX-81 microscope system and SlideBook 6.0.22 digital microscopy software (3i) for data collection and digitization. ZsGreen, mVenus, or YFP fluorescence was used to identify transfected cells, and the intensity of Alexa Fluor-594 staining for each transfected cell was measured. After background subtraction, the intensity of each cell was normalized to the mean intensity of the control group. Each experiment was repeated the number of times indicated in the figure legends.

### Caspase-Dependent Apoptosis Measurements

Apoptosis was initiated in differentiated PC12 cells by exchanging the differentiation media with CO_2_ independent media (Thermo Fisher Scientific catalog no. 18045-088) without sera, supplemented with 1 μM staurosporine (Sigma-Aldrich catalog no. S6942) to enhance the rate of apoptosis and 2 μM CellEvent caspase-3/7 Green Detection Reagent (Thermo Fisher Scientific catalog no. C10723) to assess apoptosis. Images were taken at the indicated intervals using uniform exposure settings with an Olympus IX-81 microscope system and SlideBook 6.0.22 digital microscopy software (3i) for data collection and digitization. mCerulean fluorescence was used to identify transfected cells and the intensity of Caspase-3/7 Green Detection Reagent staining for each transfected cell was measured. Transfected cells with green intensity above background were identified as apoptotic. For each experiment, the control, +S12 and +S12_AAA_ groups were measured in parallel. Three independent experiments were performed for each group.

### Western Blot

Conditioned media from cells co-transfected with pαH-S-RRAR and mVenus C1 or transfected with mVenus C1 alone were collected after 5 days expression. 15 μL of conditioned media from each group was subjected to electrophoresis on a 10% Laemmli SDS–PAGE gel. Proteins were then transferred to a 0.2 μm PVDF membrane at 100 mA overnight in Tris-glycine, pH 9.2, 20% methanol at 4 °C. The membrane was then incubated in blocking buffer (5% bovine serum albumin in Tris buffered saline supplemented with 0.1% Tween-20 (TBST)) for 1 h at room temperature with gentle rocking. The membrane was then incubated with the primary antibody (SARS-CoV-2 spike protein (RBD) polyclonal antibody (Thermo Fisher, catalog no. PA5-114451; RRID: AB_2890581) diluted 1:250 in blocking buffer) overnight at 4 °C with gentle rocking. After washing 4× in TBST for 5 min each, the membrane was incubated with the secondary antibody (goat anti-rabbit IgG HRP linked (Cell Signaling Technology catalog no. 7074; RRID: AB_2099233), diluted 1:2000 in blocking buffer) overnight at 4 °C with gentle rocking. After washing 4× in TBST for 5 min each, the membrane was developed with SuperSignal West Pico Plus chemiluminescent substrate (Thermo Fisher catalog no. 34577) according to the manufacturer’s instructions.

### RIC3 Pulldowns by S12 or S12_AAA_

The His-tags in the S12 and S12_AAA_ constructs were used for RIC3 pulldowns from lysates of three cell groups: (1) control, co-transfected at a 1:1 ratio with pLX304-RIC3 and mVenus C1; (2) +S12, co-transfected at a 1:1 ratio with pLX304-RIC3 and pαH-S-RRAR; and (3) +S12_AAA_, co-transfected at a 1:1 ratio with pLX304-RIC3 and pαH-S-AAA. HEK293T/17 cells were transfected as described above in T25 flasks and harvested after 36 h expression. All subsequent procedures were carried out at 4 °C. The cells were washed with DPBS and then lysed with 200 μL of lysis buffer (20 mM Tris, pH 8, 137 mM NaCl, 10% glycerol, 1% TX100, 2.5 U/mL benzonase (Sigma catalog no. 70746) and HALT protease inhibitor (Thermo Scientific catalog no. 87785)). After 1 h of incubation, the lysate was collected and centrifuged (20K × *g*) for 10 min to collect solubilized proteins. 50 μL of a 50% slurry of NiNTA resin (ProteinArk catalog no. Super-NiNTA10) was added to each group and incubated 2 h with inversion. The resin was then collected and washed 4× with 40 mM imidazole to remove nonspecific binding before being eluted with 50 μL of 500 mM imidazole in 1× SDS–PAGE sample buffer. 10 μL from each group was subjected to electrophoresis on a 10% Laemmli SDS–PAGE gel and a Western blot performed as described above for detecting S12 protein. RIC3 pulled down by binding to S12 was detected on the same blot using RIC-3 1:100 (G-8) antibody (Santa Cruz catalog no. sc-377408) as a primary and m-IgG2b BP-HRP 1:1000 antibody (Santa Cruz catalog no. sc-542741) as a secondary antibody.

### Spike HexaPro Protein Expression and Purification

Spike HexaPro protein was expressed in Expi293 GnTI- cells (female, human fetal kidney, RRID: CVCL_B0J7) grown in baffled flasks with Expi293 expression medium (Gibco catalog no. A14351-01) at 37 °C, 120 rpm, and 8% CO_2_ in a humidified atmosphere. Expi293 cells were transfected as follows: cells were diluted to 2.5 × 10^6^ cell/mL and grown 20–24 h. SARS-CoV-2 S HexaPro (RRID: Addgene_154754, 1.5 μg/mL final culture volume) was diluted in 1/20 final culture volume with media. Linear polyethylenimine MW 2500 (Polysciences catalog no. 23966-1, 4.5 μg/mL final culture volume) was diluted in 1/20 final culture volume with media. The DNA and polyethylenimine solutions were combined and incubated at room temperature 30 min before being added to the overnight culture with a final cell density of 2.5 × 10^6^ cell/mL. The culture was then grown overnight before stimulating protein production with the addition of 2.2 mM valproic acid (Thermo Fisher catalog no. A12962). Cells were grown for an additional 4 days. Then, the conditioned media containing Spike HexaPro protein were harvested and filtered before purification. The S12 HexaPro construct has an N-terminal 2xStrep tag. Thus, the protein solution was loaded onto a 5 mL StrepTrap XT column (Cytiva catalog no. 29401322) and then washed with 50 mM Tris, pH 8, and 150 mM NaCl. S12 was eluted with 50 mM biotin (Alfa Aesar catalog no. A14207.09), yielding ∼25 mg of high-purity S12 per liter of culture media. Trimeric S12 was isolated using size exclusion chromatography with a Superose 6 Increase 10/300 GL column (Cytiva catalog no. 29-0915-96) equilibrated with 20 mM Tris, pH 8, and 150 mM NaCl, resulting in ∼12 mg of trimeric S12 per liter of culture media. The quality of the protein was evaluated as shown in Figure S1b.

### Cryo-EM

Cryo-EM was performed using a Titan Krios cryoelectron microscope equipped with a Falcon 3 direct electron detector in the cryo-EM facility at the University of Pittsburgh School of Medicine. Briefly, Quantifoil 1.2/1.3 Au 300 mesh grids were glow discharged for 30 s at 25 mA. 3 μL of S12 (0.5 mg/mL) was applied to the grids and blotted for 3–4 s followed by plunge freezing into liquid ethane using a Vitrobot Mark IV. Data were collected on the Titan Krios using EPU at 0.832 Ans/pixel. Approximately 100 micrographs were collected, drift corrected, and autopicked using the blob picker in CryoSPARC 6. Particles were sorted using two rounds of 2D classification followed by homogeneous refinement with C3 symmetry in CryoSPARC using a low-pass filtered volume of EMD-11334 as the initial reference.

### Sequence Homology and Alignment

Sequence homology between α7nAChR (P36544) and S12 (P0DTC2) (Figure 6) was searched using the National Center for Biotechnology Information Web server Global Align (Needleman-Wunsch) algorithm (https://blast.ncbi.nlm.nih.gov/Blast.cgi) with default settings.^72^ Multiple sequence alignment of nAChR subtypes (Figure S4) was performed using the UniProt Web server Align (Clustal Omega) algorithm (https://www.uniprot.org/align) with default settings.^73^

### Quantification and Statistical Analysis

The total number of cells analyzed from each experimental group and the number of times each experiment was repeated are indicated in the figure legends. Significance was determined by two-tailed unpaired *t* test or one-way ANOVA with Dunnett’s multiple comparisons using Prism 9.4.0 software (GraphPad). A value of *p* < 0.05 was considered significant.

## Data Availability

All data generated during this study are included in this published article or its Supporting Information. The source data underlying Figures 1–7 and Figures S1–S3 are provided as Supporting Information.

## Supporting Information Available

Results showing that recombinant S12 (HexaPro) added to extracellular media (Figure S1) and extracellular S12 secreted by cells expressing S12 (Figure S2) did not suppress α7nAChR surface expression; data showing higher expression of S12_AAA_ than S12 in HEK293T/17 cells coexpressing α7nAChR (Figure S3) and the sequence alignment of the α7nAChR segment (residues 411 and 418) with α4β2nAChR and other nAChR subtypes (Figure S4) (PDF)
Source data underlying Figures 1–7 and Figures S1–S3 (XLSX)
