# Supplementary material for: SARS-CoV-2 Spike Protein Downregulates Cell Surface α7nAChR through a Helical Motif in the Spike Neck
Source: ACS Chem Neurosci. 2023 Feb 6;14(4):689–98. doi: 10.1021/acschemneuro.2c00610 (PMC9923440; doi:10.1021/acschemneuro.2c00610)
Supplement: Supplementary file 1 — cn2c00610_si_001.pdf [file cn2c00610_si_001.pdf]

Supporting Information for Publication

SARS-CoV-2 Spike Protein Downregulates Cell Surface α7nAChR Through a Helical Motif in the Spike Neck

Tommy S. Tillman[1], Qiang Chen[1], Vasyl Bondarenko[1], Jonathan A. Coleman[2], Yan Xu[1,2,3,4], Pei Tang[1,3,5*]

[1]Department of Anesthesiology and Perioperative Medicine, University of Pittsburgh, Pittsburgh, PA 15260, USA

[2]Department of Structural Biology, University of Pittsburgh, Pittsburgh, PA 15260, USA

[3]Department of Pharmacology and Chemical Biology, University of Pittsburgh, Pittsburgh, PA 15260, USA

[4]Department of Physics and Astronomy, University of Pittsburgh, Pittsburgh, PA 15260, USA;

[5]Department of Computational and Systems Biology, University of Pittsburgh, Pittsburgh, PA 15260, USA

[*]Further information and requests for resources and reagents should be directed to and will be fulfilled by the lead contact, Pei Tang (email: ptang@pitt.edu).

Supplementary Figures 1- 4

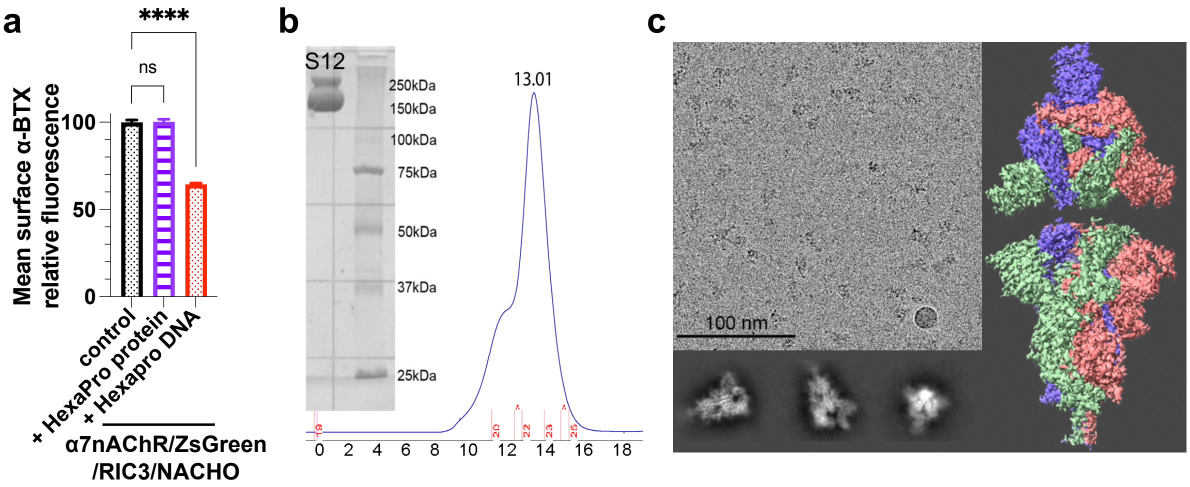

Figure S1. Recombinant S12 (HexaPro) in extracellular media did not suppress α7nAChR surface expression. (a) Relative mean intensities of α-bungarotoxin (α-BTX) labeling of transiently transfected HEK293 cells co-expressing α7nAChR/ZsGreen/RIC3/NACHO that were supplemented with 400 nM trimeric HexaPro protein in the media (purple) or co-transfected with HexaPro DNA (red). Purified HexaPro in the media for 4 hours had no effect, though co-expression of HexaPro showed a ~36% suppression of functional α7nAChR compared to the control group. Data collection was performed ~36h after transfections. Data for each group were from 2 independent experiments with a total n = 1064 (control), n=978 (+ HexaPro protein) and n=1009 (+HexaPro DNA) cells. Data are means ± SEM. p values were generated from one-way ANOVA with Dunnett's multiple comparisons; $p > 0.05$ (ns), $p \leq 0.0001$(****). **(b)** High purity HexaPro shown in the 10% SDS-page gel (left) and a SEC purification trace (right) showing isolated trimeric HexaPro with a Superose 6 Increase 10/300 GL column equilibrated with 20 mM Tris pH8 and 150 mM NaCl. S12 was expressed in Expi293F cells with Expi293F expression medium (0.5 L for each expression) for 5 days. Supernatant of the cell culture containing HexaPro was harvested and filtrated before loading onto a 5 mL StrepTrap XT column (Cytiva), which was then washed with 50 mM Tris pH 8 and 150 mM NaCl. S12 was eluted with 50 mM biotin, yielding ~25 mg of high-purity S12 per liter of culture media. (**c**) Cryo-EM demonstration of trimeric HexaPro. Representative electron micrograph (Left top) and class averages (left bottom) of S12 particles on a freshly glow discharged Quantifoil 1.2/1.3 300 mesh Au carbon grid. The top and side views of the electron density map (right) generated from 34,317 particles, showing characteristic trimeric structural features of S12. The density map fits the closed conformation of S12 (PDBID: 6ZWV) with CC_mask=0.73.

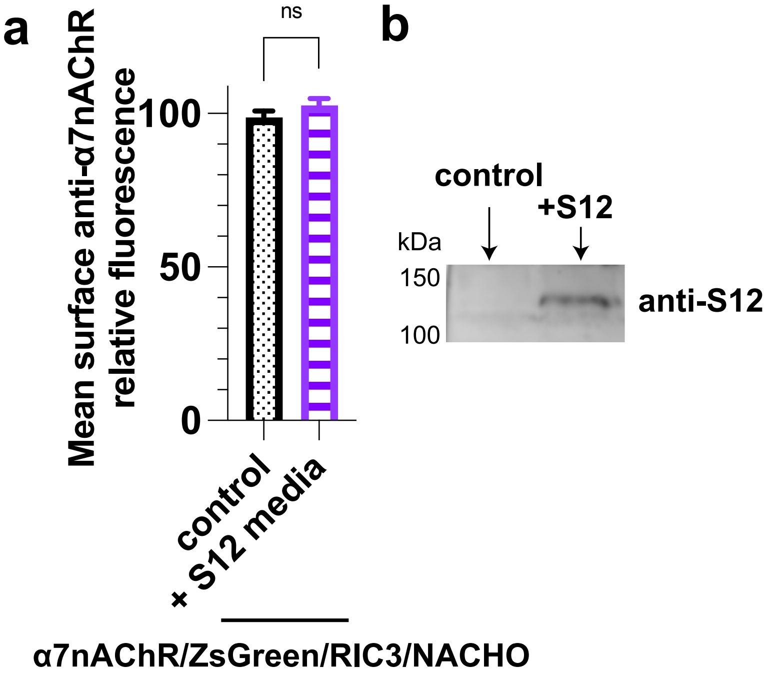

Figure S2. S12 in extracellular solution does not suppress α7nAChR surface expression. (a) Relative mean intensities of anti-α7nAChR immunofluorescent staining of transiently transfected HEK293 cells co-expressing α7nAChR/ZsGreen/RIC3/NACHO show no significant difference in surface α7nAChR expression in the absence (control) or presence (+S12 media) of conditioned media replacement from cells expressing S12 (replaced 3 times over a 24-hour period). Data collection was performed ~36h after transfections. Data were from n = 1563 (control) and n = 1306 (+S12 media) cells. p value is from two-tailed unpaired t test; $p > 0.05$. Data are means ± SEM. **(b)** Western blot showing secreted S12 from HEK293 transfected with cDNA encoding S12. The blot was stained using anti-SARS-CoV-2 Spike Protein (RBD) antibody (1:250 PA5-114451 Invitrogen) with 15 µL conditioned media from each group. A band consistent with S12 (~142 kDa) was detected only in the +S12 group.

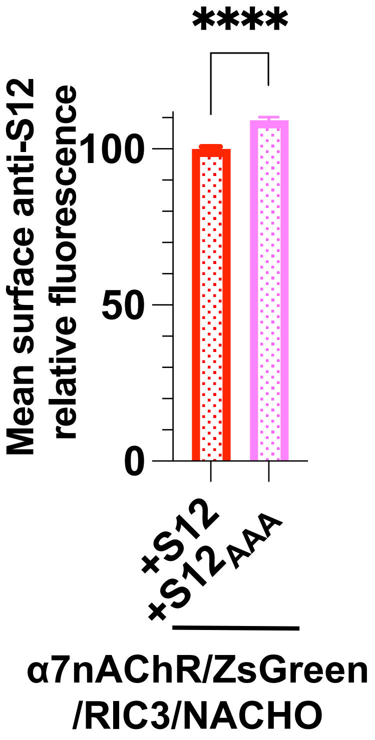

Figure S3. $S12_{AAA}$ expression is ~9% greater than S12 expression in HEK293T/17 cells co-expressing α7nAChR. Summary graph showing relative mean intensities of immunofluorescent staining of transiently transfected HEK293T/17 cells co-expressing α7nAChR/ZsGreen/RIC3/NACHO with either S12 (+S12) or $S12_{AAA}$ (+$S12_{AAA}$). Total expression of S12 and S12AAA were detected in permeabilized cells using SARS-CoV-2 Spike Protein (RBD) Polyclonal Rabbit Antibody (1:250 PA5-114451 Invitrogen). Data collection was performed ~36 h after transfections. Data is from n = 7777 (+S12) and n = 5720 (+$S12_{AAA}$) cells. $p \leq 0.0001$(****) from two-tailed unpaired t test. Data are mean ± SEM.

```
ACHA7_HUMAN(P36544) 411 LAKILEEV 418
ACHA4_HUMAN(P43681) 536 LTRAVEGV 543
ACHB2_HUMAN(P17787) 399 LREAVDGV 406

ACHA2_HUMAN(Q15822) 416 MQKALEGV 423
ACHA3_HUMAN(P32297) 410 IKEAIQSV 417
ACHA5_HUMAN(P30532) 356 LEAALDSI 363
ACHA6_HUMAN(Q15825) 404 VEDVINSV 411
ACHA9_HUMAN(Q9UGM1) 396 YKVLTRNI 363
ACHB4_HUMAN(P30926) 403 VQEALEGV 406
```

Figure S4. Sequence alignment of the α7nAChR segment (residues 411 and 418) with α4β2nAChR and other nAChR subtypes. The protein sequences of α7, α2, α3, α4, α5, α6, α9, β2, and β4 subunit of human nicotinic acetylcholine receptors were obtained from UniProt and aligned using the UniProt webserver Align (Clustal Omega) algorithm (https://www.uniprot.org/align) with default settings.[1] Note that smaller size hydrophobic residues have occupied the position equivalent to 414Ile in α7nAChR, such as 539Ala in α4 and 402Ala in β2nAChR.

References

(1) Sievers, F.; Higgins, D. G. Clustal Omega for making accurate alignments of many protein sequences. *Protein Sci* **2018**, *27* (1), 135-145. DOI: 10.1002/pro.3290
